# The cost-effectiveness in the use of HIV counselling and testing-mobile outreaches in reaching men who have sex with men (MSM) in northern Nigeria

**DOI:** 10.7448/IAS.17.4.19610

**Published:** 2014-11-02

**Authors:** Chiedu Ifekandu, Aliyu Suleiman, Ogechukwu Aniekwe

**Affiliations:** 1 HIV/Key Populations, Population Council, Abuja, Nigeria; 2 HIV/Key Populations, World Health Organization (WHO), Northern Nigeria Region, Nigeria; 3 HIV/Key Populations, African Health Project (AHP), Abuja, Nigeria

## Abstract

**Introduction:**

Men who have sex with men (MSM) are at increased risk of HIV and other STI infections in Nigeria. This is because MSM are afraid to seek medical help because the healthcare workers in various facilities are afraid of the consequences if they provide services for MSM citing the law as a reason not to intervene. MSM in northern states of Nigeria are facing double-jeopardy because the few international partners working in MSM in Nigeria are pulling out of these volatile areas because of the fear of attacks by the Boko Haram and the Nigerian law enforcement agencies.

**Objectives:**

The intervention was conducted to promote affordable and sustainable HIV care and treatment for MSM in Nigeria.

**Methods:**

This intervention was conducted in the Boko Haram ravaged cities of Kano and Maiduguri (North-East Nigeria). Twenty MSM-key influencers from the two cities were identified and trained on HIV counselling and testing, caregivers, case managers and on initiation process for ARV treatment for new HIV+MSM as well as ethical considerations.

**Results:**

The mean age of the key influencers was 24 years +/−SD. Each of the trained 20 key influencers reached 20 MSM-peer with condom promotion, HCT, referral to identified MSM-community health centers and follow-up/caregiving within the space of one month. The project was able to reach 400 MSM in the two cities. 89% of the peers consented to HCT. HIV prevalence among the participants was at 18%. The project recorded ARV-successful referral to healthcare facilities for the respondents that tested positive. The key influencers have been following up for ARV-adherence.

**Conclusions:**

Use of community members should be promoted for sustainability and ownership. It also helps in eradicating socio-cultural barrier to HIV intervention for MSM. Moreover, this proves to be one of the safest and affordable methods of reaching MSM in Nigeria in this ugly time of legalization of homophobia in the country's constitution.

